# Can mesenchymal stem cell lysate reverse aging?

**DOI:** 10.18632/aging.101595

**Published:** 2018-10-24

**Authors:** Ming-Fen Hsu, Szu-Hsien Yu, Sheng-Ju Chuang, Tom Kwang-Chun Kuo, Pawan K. Singal, Chih-Yang Huang, Chung-Lan Kao, Chia-Hua Kuo

**Affiliations:** 1 Laboratory of Exercise Biochemistry, University of Taipei, Taipei, Taiwan; 2 Université Catholique de Louvain and de Duve Institute, Brussels, Belgium; 3 Stem Cell Research Center, National Yang-Ming University, Taipei, Taiwan; 4 Institute of Cardiovascular Sciences, St Boniface General Hospital Research Centre and Department of Physiology and Pathophysiology, Faculty of Medicine, University of Manitoba, Winnipeg, Canada.; 5 Department of Health and Nutrition Biotechnology, Asia University, Taichung, Taiwan; 6 Graduate Institute of Basic Medical Science, China Medical University, Taichung, Taiwan; 7 Department of Physical Medicine and Rehabilitation, Taipei Veterans General Hospital and National Yang Ming University, Taipei, Taiwan; * Equal contribution

**Keywords:** longevity, osteopenia, lipopenia, paracrine effect, bone loss, glucose, lifespan

## Abstract

Recent findings regarding uses of adipose-derived mesenchymal stem cell (MSC)-lysate on weight loss and improved glucose tolerance in mice on a high-fat diet suggest an encouraging possibility of using MSC lysate for an anti-aging intervention in humans. However, weight loss and lipopenia during late life can be as life-threatening as hyperglycemia during early adulthood. For this 3-year lifelong experiment, a total of 92 rats were randomized into the vehicle-injected group (F=22; M=24) and the MSC lysate injected group (F=22, M=24). We examined longevity, spontaneous locomotor activity, and body composition in rats maintained on a normal diet and received an intermittent treatment of human adipose-derived MSC lysate (3 times a week, 11 times a month given every second month), starting at 12 months of age until natural death. In substantiating previous knowledge regarding the effects of long-term MSC lysate treatments on fat loss and insulin resistance, the present findings also highlighted a shortened average lifespan, a longer inactive time, and a greater bone loss with a relative increase of lean mass in MSC lysate rats with respect to controls. Conclusion: Our data suggest that MSC lysate treatments stimulate disparity in tissue development and produce a cachexia-like effect to decrease longevity.

## Introduction

Mesenchymal stem cells (MSC) has high potency to differentiate into osteoblasts, chondroblasts, adipocytes and myoblasts [1–3] with an advantage of hypoimmunogenic property for allogenic applications across different individuals and species [4,5]. Human adipose-derived MSC has been shown to have the same effect as mice adipose-derived MSC [6] on preventing weight gain and glucose intolerance in mice on a high fat diet [7,8]. The therapeutic benefit of MSC transplantation is thought to be mediated by its paracrine effect [9–11]. Compared to cell transplantation, lysate from human adipose-derived MSC has demonstrated a comparable improvement in glucose tolerance in mice on a high-fat diet [8]. Since overweight and insulin resistance are regarded as complications of the aging process, aforementioned studies suggest a possibility of using adipose-derived MSC lysate for anti-aging intervention.

However, mediators released by MSCs are also known to indistinguishably stimulate both normal cells as well as the mutated cells that gain greater capability to grow [12,13]. In a multicellular organism, somatic mutated cell population accumulates due to numerous cell division during size expansion and cell turnover [14]. Thus, the present study aimed to ascertain the effects of long-term MSC lysate treatment on longevity, activity level, and body composition of rats fed with standard diet. Evolutions of body fat, bone, and lean mass of male rats were assessed by dual energy X-ray absorptiometry (DEXA) at 19 months and 27 months of age. Based on the theoretical framework, we expected increases in longevity and vitality of naturally aging rats after prolonged MSC lysate treatment.

## RESULTS

**Figure 1:**
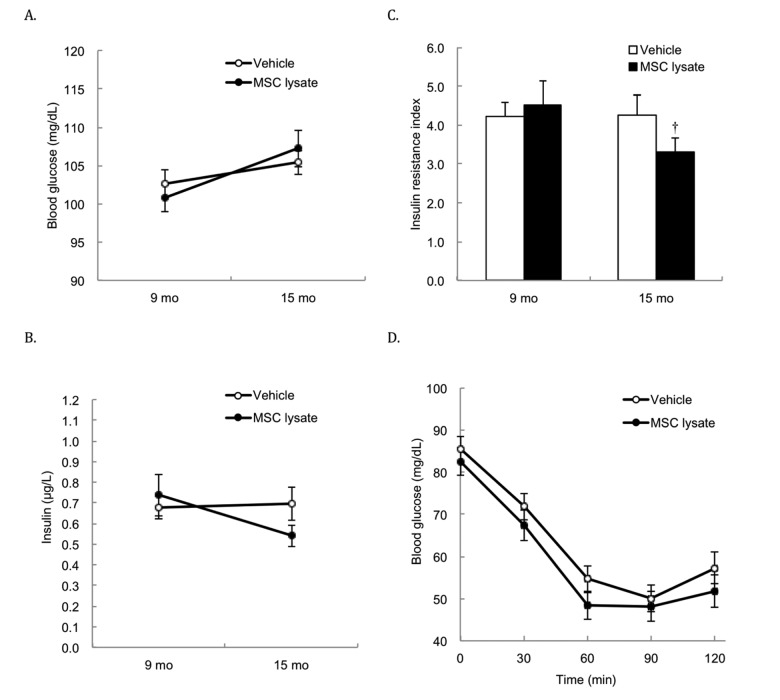
**Fasting glucose and insulin in blood**. Survivors until 15 months of age were included for the Pre-Post comparison (Vehicle: N = 43; MSC lysate: N = 39), measured at 9 months and 15 months of age. Differences in glucose (**A**) and insulin (**B**) between the Vehicle and MSC lysate groups did not reach statistical significance after 2 sessions of MSC lysate treatment. However, insulin resistance index (product of fasting glucose and insulin) was moderately decreased in the MSC lysate group, but no change was observed in the Vehicle group (**C**). Blood glucose concentrations during insulin tolerance test (insulin injection at 0.3 U/kg body weight) of the MSC lysate group was marginally lower than those of the Vehicle group, after 5 sessions of MSC lysate treatment (Vehicle: N = 27; MSC lysate: N = 24) (main effect of treatment of two-way ANOVA: *p* = 0.06) (**D**). † Significant difference against pre-treatment value (9 months of age), *p* ≤ 0.05. Data for male and female rats are presented separately in Supplementary figures (Figure S1_Male and S1_Female). Abbreviation: MSC, adipose-derived mesenchymal stem cell.

Table 1 shows cytokine profile (relative concentration) of human adipose-derived MSC lysate used in the study. Three months after the treatment (2 bimonthly sessions of intervention), both glucose (Figure 1A) and insulin (Figure 1B) of rats were not significantly different between the Vehicle and MSC lysate groups. The insulin resistance index (homeostatic model assessment-insulin resistance, HOMA-IR) (Figure 1C) of the MSC lysate group was significantly lower than that of the Vehicle group at 15 months of treatment (*p* ≤ 0.05, Cohen's d = 0.35). Glucose concentrations during insulin tolerance test in the MSC-injected group during the insulin tolerance test were marginally lower than those in the Vehicle group (*p* = 0.06, main effect) (Figure 1D). For the female rats, glucose levels during insulin tolerance test in the MSC lysate group were lower than those in the Vehicle group (Figure S1_Female) at 21 months of age (*p* ≤ 0.05, Cohen's d = 0.57). This difference was not observed in the male rats. For the male rats, fasting insulin reduced by ~40% with a slight glucose increase in the MSC lysate group (*p* ≤ 0.05, Cohen's d = 0.70), whereas no change was found in the Vehicle group (Figure S1_Male).

**Table 1:** Cytokine profile in the MSC lysate. Data are presented as relative concentrations (signal intensity among array images).

bFGF 170.2	MCP-1 34.2	G-CSF 21.8	Leptin 17.5	IGF-I 15	TIMP-4 11.3	BMP-4 9	SDF-1b 6.2	E-Selectin 4
GRO 89.5	AgRP 33.8	TNF-β 21.7	FGF-6 17.5	EGF 14.8	Tie-2 11.3	Leptin R 8.8	Flt-3L 5.9	CXCL-16 4
IL12-p40 81.2	VEGF 32.9	IL-2 Ra 21.5	LIGHT 17.4	SCF R 14.7	MIP-1-∂ 11.2	FGF-7 8.5	IL-7 5.2	CX3CL1R 3.9
FGF-9 81.2	MSP-a 32.9	MIP-3-β 21.3	IL11 16.8	ICAM-1 14.7	HCC-4 11.1	IGFBP-2 8.5	MMP-9 5.1	ICAM-2 3.7
MIF 80.5	VEGF-D 32	NT-3 21.1	IL-1 RI 16.8	HGF 14.7	CKβ8-1 11.1	CD144 8.5	MMP-13 5.1	CD14 3.7
TIMP-2 59.6	BTC 30.8	RANTES 21	sTNF-RI 16.6	Eotaxin-3 14.6	LIF 10.7	Siglec-5 8.5	FasL 5.1	MPIF-1 3.4
MIP-1-α 59.6	ALCAM 29.1	IL-1ra 20.8	MIG 16.4	I-TAC 14	Tie-1 10.5	PECAM-1 8.5	ErbB3 5.1	L-Selectin 3.4
IGF-BP-3 59.4	LAP 27.7	IL12-p70 20.8	GITR 16.3	SCF 13.6	MCP-4 10.4	PDGF-AB 8.5	M-CSF R 4.8	BMP-7 3.1
FGF-4 58	IGF-BP-6 27	MDC 20.4	SDF-1 16.1	β-NGF 13.5	IL-16 10.3	CTACK 8.3	CT-1 4.8	ICAM-3 2.8
TNFRSF6 57.3	MIP-1-β 26	GITR-L 19.6	TGF b2 16.1	EGF-R 13.5	GCP-2 10.3	VEGF R2 8.2	BMP-5 4.8	IL-18 BPa 2.8
TIMP-1 50.9	OGP 25.1	TGF-β3 19.4	IL-18 Rb 15.5	MMP-1 13.3	CNTF 10.3	IL-9 7.6	dtk 4.7	BMP-6 2.7
TRAIL-R4 43.3	NT-4 24.6	TRAIL-R3 19.2	sTNF RII 15.4	IL-13 Ra2 13.3	GDNF 10.2	TECK 7.5	IL-2 Rb 4.5	NGF R 2.5
PDGF Rb 41	sgp130 23.2	IL8 18.9	TARC 15.4	IGF-I SR 12.5	TGF-a 10.2	PDGF Ra 7.3	IL-5 4.5	IL-5 Ra 2.5
Ang-2 38.8	PlGF 23	IL-6 18.4	I-309 15.3	IFN-γ 12.3	Prolactin 10.2	CD80 7.1	IL-4 4.5	Endoglin 2.3
uPAR 38.3	LIF/OSM 22.7	TGF-β1 18.2	Eotaxin-2 15.3	MCP-2 12.1	AREG 9.5	IL-15 7	IL-10 4.5	DR6 2.3
BDNF 37.7	TNF-α 22.5	IL-1β 18.2	PDGF-AA 15.3	MIP-3-α 11.8	PARC 9.3	MCP-3 6.6	Eotaxin 4.5	Avtivin A 2.3
TPO 36.7	IL-6R 22.2	axl 18.1	IP-10 15.3	IL-1α 11.6	Acrp30 9.2	Ang 6.6	CCL28 4.3	IL-2 Rg 2
IGF-II 36.2	M-CSF 22	IL-1 R4 18	IL17 15.1	ENA-78 11.4	PDGF-BB 9.2	IL-2 Ra 6.5	BLC 4.1	IL-1 R II 2
IL-3 35.6	GRO-α 21.8	VEGF R3 17.8	NAP-2 15.1	ENA-78 11.4	IL-2 9	IGFBP-1 6.5	IL-10 Rb 4	IGFBP-4 1.9

Abbreviation: MSC, adipose-derived mesenchymal stem cell.

**Figure 2:**
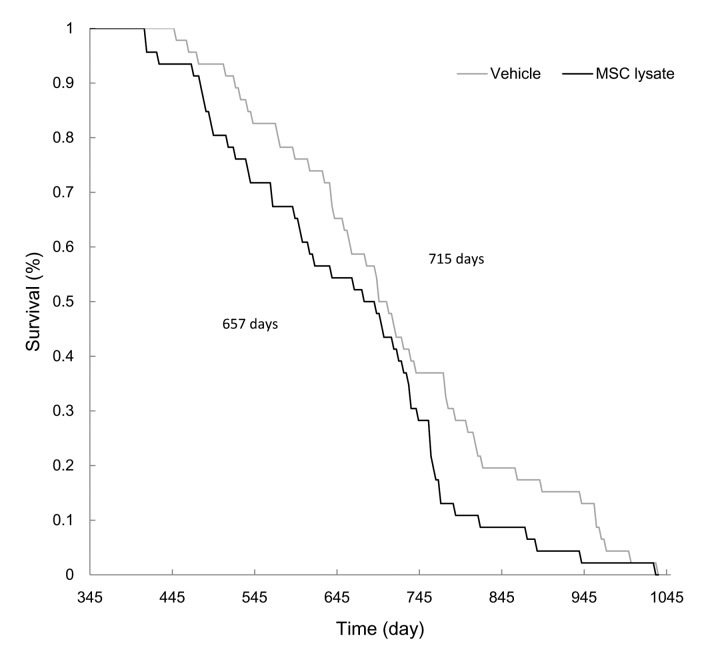
**Survival curve.** MSC lysate treatment started from 12 months of age (Vehicle: N = 46; MSC lysate: N = 46). Mean lifespans of the vehicle and MSC lysate groups were 715 d and 657 d, respectively (*p* ≤ 0.05). Data for male and female rats are presented separately in Supplementary figures (Figure S2_Male and S2_Female). Abbreviation: MSC, adipose-derived mesenchymal stem cell.

In the survival analysis (Figure 2), average longevity of the MSC-lysate group was lower than that of the Vehicle group (657 ± 21 d vs. 715 ± 22 d, *p* ≤ 0.05, Cohen's d = 0.40). Female and male rats showed similar trend in shortened longevity during lifelong MSC lysate treatment. Difference in longevity between the Vehicle and MSC lysate groups remained significant in the male rats (Figure S2_Female and Figure S2_Male) but not in the female rats.

**Figure 3:**
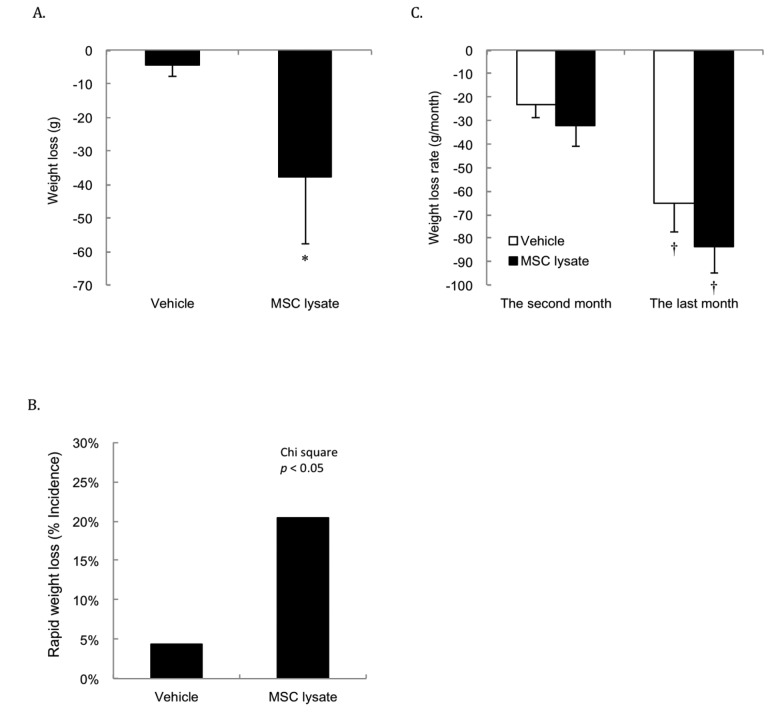
**Body weight changes.** Survivors until 15^th^ months were included for the Pre-Post comparison (Vehicle: N = 43; MSC lysate: N = 39), measured at 9 months and 15 months of age. Significant weight loss was observed in the MSC lysate group following 2 sessions of intervention in the first 3 months (*p* ≤ 0.05) (**A**). Greater incidence of weight loss (more than 7% of peak body weight during their lifetime) in the MSC lysate group (9 out of 46) than that in the vehicle group (2 out of 46) was observed (Chi-square, *p* ≤ 0.05) (**B**). Accelerated weight loss occurred 1 month before death is the common feature for aged rats regardless the treatment groups (Vehicle: N = 46; MSC lysate: N = 46) (**C**). † Significant difference against 2 months before death, *p* ≤ 0.05. Data for male and female rats are presented separately in Supplementary figures (Figure S3_Male and S3_Female). Abbreviation: MSC, adipose-derived mesenchymal stem cell.

Significant weight loss occurred in the first 3 months (2 sessions) of MSC lysate treatment (Figure 3A). Weight loss rate in the MSC-treated group was more than 4 folds of the Vehicle group during this short period (*p* ≤ 0.05, Cohen's d = 0.35). Figure 3B shows greater incidence of rapid weight loss (weight loss more than 7% of peak body weight) in the MSC lysate treated group than that in the Vehicle group in the first 3 months of the treatments (Chi-square, *p* ≤ 0.05). Rapid weight loss from 1 month before death appeared to be a common sign for both groups regardless of the treatments (Figure 3C). Significant difference in the rate of weight loss between the Vehicle and MSC lysate groups remained significant only in the male rats (Figure S3_Female and Figure S3_Male).

**Figure 4:**
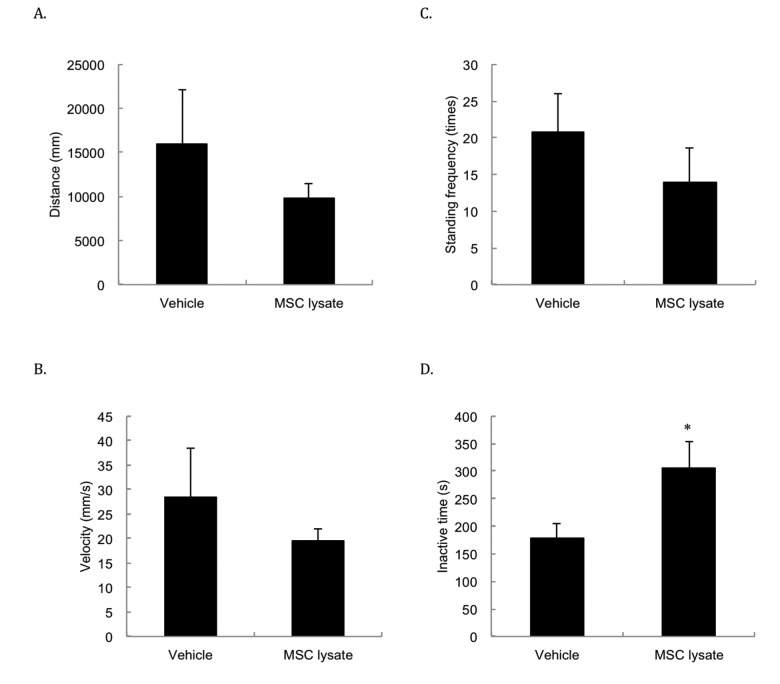
**Spontaneous locomotor activity.** Spontaneous locomotor activity was measured at 24 months of age. The differences in spontaneous movements in distance (**A**), velocity (**B**), and standing frequency (**C**) between two groups do not reach statistical significance. Inactive time (**D**) was significantly lengthened following 1-year adipose-derived mesenchymal stem cell lysate (MSC lysate) (6 sessions). * Significant difference against the Vehicle group, *p* ≤ 0.05. Abbreviation: MSC, adipose-derived mesenchymal stem cell.

Spontaneous locomotor activities (Figure 4) including moving distance (Figure 4A), velocity (Figure 4B) and standing frequency (Figure 4C) were not significantly different between the Vehicle and MSC lysate groups, but physically inactive time (Figure 4D) of the MSC-treated rats was 73% longer than that of the Vehicle group following 1 year of treatment (*p* ≤ 0.05, Cohen's d = 1.18).

**Figure 5:**
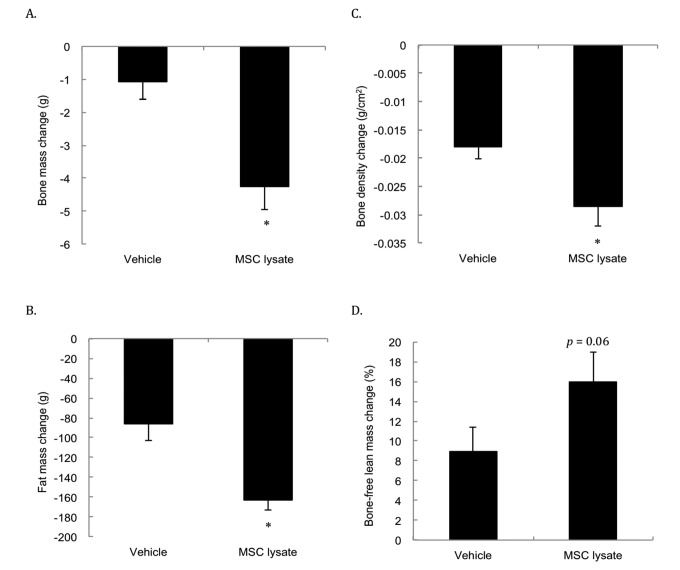
**Body composition changes during an 8-month MSC lysate intervention (between session 4 and 8 at 17^th^ and 28^th^ months of age) for male rats.** Survivors until 28 month of age were included for the Pre-Post comparison by Dual-energy x-ray absorptiometry (DEXA) analysis. Only male rats were measured due to consideration of technical limitation of the DEXA in size. Decreases in bone mass (**A**), bone density (**B**), fat mass (**C**) occurred in parallel with marginally increased bone-free lean mass (*p* = 0.06) (**D**). * Significant difference against the Vehicle group, p ≤ 0.05. Abbreviation: MSC, adipose-derived mesenchymal stem cell.

Figure 5 presents the DEXA analysis conducted at 19 months and 27 months of age (i.e., after 4 and 8 sessions of treatments). Significantly faster reductions for bone mass (Figure 5A) and bone mineral density (Figure 5B) in the MSC lysate group were observed compared with the Vehicle group. The rates of bone mass loss and bone mineral density decrease in the MSC lysate treated rats were approximately 3.9 times and 1.5 times of those in the Vehicle group, respectively (*p* ≤ 0.01 and *p* ≤ 0.05, respectively, Cohen's d = 1.19). Fat mass (Figure 5C) of the MSC lysate treated group also decreased significantly during the late stage of aging. The rate of the fat loss in the MSC lysate group was approximately 1.9 folds of that in the Vehicle group (*p* ≤ 0.01, Cohen's d = 1.0). Fat mass loss and bone mass loss occurred in parallel with a marginal increase in percent bone-free lean mass in the MSC-treated rats (Figure 5D).

## DISCUSSION

Our data with normal diet somewhat confirmed the previous studies on weight loss and improved glycemic control after human adipose MSC and MSC lysate treatments in rats under high fat diet [7,8]. However, we found a decreased longevity and an increased inactive time with the lifelong MSC lysate treatment. Although the fat loss outcome of MSC lysate treatment is considered metabolically favorable in adults during the weight rising stage of life, weight loss during the end stage of life or during illness often relates to debilitating health and increased mortality [15,16]. In humans, death rate accelerates when weight loss exceeds approximately 7% from the peak body weight during late stage of life [17,18] as well as in healthy young animals [19].

The life-shortening effect of MSC lysate could be associated with accelerated osteopenia and lipopenia. In human patients, the survival chances in the lipopenic or osteopenic elderlies are lower than non-lipopenic or osteopenic elderlies [20]. With regard to the concomitant increases in the lean mass during fat and bone losses for the MSC lysate treated rats, we would draw caution that there is a technical limitation of DEXA to distinguish muscle mass from other types of lean tissue such as fibrotic tissues, tumors, and fluid retention [21]. During a 10-y follow-up human study using DEXA for measuring body composition, an increase in central lean mass (trunk) together with losses in appendicular lean mass and visceral fat was associated with increased mortality in older individuals aged above 50 y [21,22]. Muscle mass is known to closely associate with physical vitality and survival length in aged animals and humans during the late stages of life [23]. Therefore, increased bone-free lean mass concurrent with lengthened inactive time during the MSC lysate treatment suggests that the fat-free lean tissue increase detected by DEXA is less likely to associate with increased percent muscle mass.

Therapeutic benefit of MSC transplantation is generally considered to mediate by its paracrine effect [9–11], it remained unclear how MSC-lysate injection led to a life-shortening effect with decreased vitality. In a multicellular system, clearance of aged environment (i.e. senescent cells and cell scaffolds) must be completed before onset of cell regeneration to maintain tissue health during cell turnover [24]. Simply strengthening the later process by providing signals from MSC may cause imbalance of the two processes. Thus, the preservation of lean tissues in the MSC-injected rats detected by DEXA might be associated with insufficient clearance of aged components during aging.

In contrast to unicellular organisms, multicellular organisms may be described as a cooperative society of living cells, where birth and death of cells occur continuously within the same body [25]. Increasing cell population in a multicellular system attains an advantage of sharing efforts among specialized partner cells. However, size expansion inevitable increases thermodynamic instability. Weight gain during early development increases absolute number of somatic mutations due to increased amounts of cell divisions [26]. Persistent inflammation (or non-healing wound) increases incidence of tumorigenesis [27], as a result of random genetic mutation during endless cell regeneration. Components in MSC lysate, such as FGFs, TGF-beta, VEGFs, and many other cytokines [28] participating in the regenerative phase of inflammation, may indifferently stimulate cell proliferations on both normal cells and the mutated cells that are naturally selected for high proliferation potential.

Previous studies in MSC-lysate based therapy on obese mice have provided valuable knowledge of using MSC lysate to prevent weight gain induced by high fat diet [8]. Our data on normal Sprague Dawley rats without common comorbidities that accompany older patients should be interpreted with caution when the knowledge is generalized to human patients. To definitely settle the question on whether MSC lysate is life shortening or anti-aging, more investigation should focus on optimization of dosage, frequency, and timing of MSC lysate administration at different stages of life.

## CONCLUSION

The current treatment regimen of MSC lysate, when started from middle age, does not prolong and appears to decrease longevity and vitality of normally aging rats. Disparity in development of fat, bone, and bone-free lean tissues concurrent with accelerated weight loss during the lifelong MSC lysate treatment implicates the importance of balanced tissue growth and size maintenance in sustaining a robust multicellular system.

## MATERIALS AND METHODS

### Animals and study design

This study was approved by the Animal Care and Use Committee at the University of Taipei (approval number 200901001) and conformed to the ethical rules made by the Institutional Animal Care and Use Committee (IACUC) as well as the Law of Taiwan in animal protection. Sprague Dawley (SD) rats (1 month of age) with known date of birth were obtained from BioLASCO Taiwan Corporation (Yi-Lan, Taiwan) and maintained in the Animal Center of the University of Taipei (Taipei, Taiwan). Animals were kept in a well maintained facility with the controlled temperature of 22°C, relative humidity of ~50%, 12/12 h light/dark cycle. Rats (two per cage) were provided with standard laboratory chow (PMI Nutrition International, Brentwood, MO, USA) and tap water ad libitum. This study randomized a total of 92 rats into the vehicle-injected group (F=22; M=24) and the MSC lysate injected group (F=22, M=24). Rats were intraperitoneally injected with either vehicle or MSC lysate until natural death.

### Treatment regimen

Rational of dosage: A dose-escalating study [29], tested dosages from 1,000 to 1,000,000 MSCs per kg body weight, reported dose-dependent beneficial effects of MSC on cardiac function within the range of 100,000 and 1,000,000 cells per kg body weight. In this long-term lysate study, lower limit of the dosage range corresponding to ~100,000 MSC per kg body weight was chosen. This dosage was moderately greater than a previous MSC lysate study (dosage equivalent to 70,000 cells), in which fat loss and improved metabolic syndromes in mice under high-fat diet can be observed over the course of 3 months [8].

Adipose-derived MSC lysate (20 μl/kg body weight) was delivered to rats by intraperitoneal injection, 3 times a week, 11 times a month, starting at 12 months of age until natural death. To prevent potential desensitization of the recipients to MSC lysate, delivery was made on every second month. The Vehicle group received intraperitoneal injections with the same volume of sterilized 0.9% saline. Body weight was recorded every 3 d. Fasting glucose and insulin measurements (HOMA-IR) were conducted at 15 months of age (after 2 bimonthly sessions of MSC treatment) compared with Pre (9 months of age). Insulin tolerance test was performed at 21 months of age (after 5 bimonthly sessions of MSC treatment). Spontaneous motor activity was monitored at 24 months of age (after 6 bimonthly sessions of MSC treatment).

### MSC lysate preparation and characterization

MSC lysate preparation procedure was modified from a previous study [30]. Briefly, human adipose-derived stem cells were cultured in alpha-minimal essential medium (α-MEM) supplemented with 2% fetal bovine serum and 2 mM L-glutamine, and were incubated at 37°C, 5% CO_2_. Once cells reached 80% confluency, these were harvested by enzymatic detachment using trypsin-EDTA, rinsed with 45 ml of PBS and transferred into 50 ml conical tube for centrifugation to pellet the cells. Supernatant was removed and the pellet was washed (3 times) with 50 ml of PBS with centrifugation to remove residual trypsin-EDTA and components from culture media. In order to make cell lysate, 10 ml of deionized H_2_O was added to the cell pellet and incubated at room temperature for 30 min to allow osmotic rupture of the cell membranes. The cell-free lysate was prepared by subjecting the ruptured cell pellet to 3 freeze-thaw cycles to further dissociate the lysed cell sediments, followed by centrifugation at 1000 g to remove insoluble materials. Four separate batches of MSC lysate were pooled together for intervention. The content of the lysate was assayed using RayBio® C-Series Human Cytokine Antibody Array Kit according to the manufacturer’s instructions (Raybiotech Northcross, GA, USA).

### Glucose, insulin and insulin tolerance test

Insulin resistance occurs during middle age. Therefore, HOMA-IR was measured at 15 months of age. Fasting blood glucose of rats (15 months of age) was always measured immediately after sample collection using Accu-chek® performa system (Roche Diagnostics, Indiana, USA). Plasma insulin was measured using enzyme-linked immunosorbent assay (ELISA) kit (Mercodia, Mercodia AB, Uppsala, Sweden). After 12 h fasting, blood was collected from tail into EDTA-contained tube and plasma was obtained after centrifugation at 4°C, 3000 rpm for 10 min. Optical density was read at 450 nm using ELISA reader (Tecan GENios, A-5082, Austria) and insulin level was expressed as μg/L. Insulin resistance index was calculated using fasting blood glucose and insulin level by a formula presented earlier [31].

To confirm whether the HOMA-IR results at 15 months of age is associated with insulin sensitivity, insulin tolerance test was also measured at 21 months of age. For the test, rats (21 months of age) were injected (I.P.) with insulin (0.3 U/kg BW, Humulin R, Eli Lilly, Indianapolis, Indiana, USA) following 12 h fasting. Glucose (mg/dL) from tail blood was measured before and 30, 60, 90 and 120 min after insulin injection.

### Spontaneous locomotor activity

Since loss of physical vitality is a main concern during the end stage of life, spontaneous locomotor activity was monitored at 24 months of age, using Locoscan system (Clever Sys, VA, USA). Rats were moved from their housing cage to the testing cage (40 cm^3^ black plastic custom cage) before activity assessment. One week prior to the assessment, rats were placed in the testing cage for 10 min daily for acclimation. Spontaneous activity was performed in a quiet and dark environment. Rats were placed into the center of testing cage and free to move. A digital camera with infrared light source mounted above the center of testing cage to record video on their physical activity for 20 min. The first and last 5 min periods were eliminated from analysis to avoid disturbances related to human access to dark room. Ten min videos of the middle time frame were analyzed, which detected rats movements based on video-tracking of multiple individual body parts, posture and frequency of movements. Activity parameters included traveled distance (mm), velocity (mm/s), vertical standing frequency (times) and inactive time (s).

### Body composition

Body composition of rats was measured by DEXA (Lunar iDXA, GE Medical Systems, WI, USA) with small animal software package. DEXA scans were performed under isofluorane anesthesia after 12-h fasting. Body composition data included bone mass (gram), bone density (g/cm2), fat mass (gram), percentage body fat (%), bone-free lean mass (gram), percent bone-free lean mass (%). DEXA analysis was conducted twice at 19 months and 27 months of age. Only male rats were measured due to technical limitation of accurate DEXA analysis for small size rats among some female rats.

### Statistical analysis

Independent *t* test was used to compare mean difference of all variables between Vehicle and MSC lysate groups. Pair t test was used to compare the mean differences between first and second DEXA measurements among paired survivors. Chi-square was used to compare the difference in incidence of weight loss (7%) between groups during treatments. Two-way ANOVA was used to determine the main and interactive effect for insulin tolerance test. Cohen’s d was used to determine between-group effect sizes for dependent variables. Probability of type I error was set at 5% or less for significance. All results were expressed as mean ± standard error of the mean (SEM).

## Supplementary Material

Supplementary Figures

## References

[1] Beresford JN, Bennett JH, Devlin C, Leboy PS, Owen ME. Evidence for an inverse relationship between the differentiation of adipocytic and osteogenic cells in rat marrow stromal cell cultures. J Cell Sci. 1992; 102:341–51. 1400636 10.1242/jcs.102.2.341

[2] Berry MF, Engler AJ, Woo YJ, Pirolli TJ, Bish LT, Jayasankar V, Morine KJ, Gardner TJ, Discher DE, Sweeney HL. Mesenchymal stem cell injection after myocardial infarction improves myocardial compliance. Am J Physiol Heart Circ Physiol. 2006; 290:H2196–203. 10.1152/ajpheart.01017.2005 16473959

[3] Caplan AI. Mesenchymal stem cells. J Orthop Res. 1991; 9:641–50. 10.1002/jor.1100090504 1870029

[4] Griffin MD, Ritter T, Mahon BP. Immunological aspects of allogeneic mesenchymal stem cell therapies. Hum Gene Ther. 2010; 21:1641–55. 10.1089/hum.2010.156 20718666

[5] Ra JC, Shin IS, Kim SH, Kang SK, Kang BC, Lee HY, Kim YJ, Jo JY, Yoon EJ, Choi HJ, Kwon E. Safety of intravenous infusion of human adipose tissue-derived mesenchymal stem cells in animals and humans. Stem Cells Dev. 2011; 20:1297–308. 10.1089/scd.2010.0466 21303266

[6] Cao M, Pan Q, Dong H, Yuan X, Li Y, Sun Z, Dong X, Wang H. Adipose-derived mesenchymal stem cells improve glucose homeostasis in high-fat diet-induced obese mice. Stem Cell Res Ther. 2015; 6:208. 10.1186/s13287-015-0201-3 26519255 PMC4628312

[7] Ji AT, Chang YC, Fu YJ, Lee OK, Ho JH. Niche-dependent regulations of metabolic balance in high-fat diet-induced diabetic mice by mesenchymal stromal cells. Diabetes. 2015; 64:926–36. 10.2337/db14-1042 25277392

[8] Lee CW, Hsiao WT, Lee OK. Mesenchymal stromal cell-based therapies reduce obesity and metabolic syndromes induced by a high-fat diet. Transl Res. 2017; 182:61–74.e8. 10.1016/j.trsl.2016.11.003 27908750

[9] Zhang H, Qiu X, Shindel AW, Ning H, Ferretti L, Jin X, Lin G, Lin CS, Lue TF. Adipose tissue-derived stem cells ameliorate diabetic bladder dysfunction in a type II diabetic rat model. Stem Cells Dev. 2012; 21:1391–400. 10.1089/scd.2011.0244 22008016 PMC3359635

[10] Volarevic V, Arsenijevic N, Lukic ML, Stojkovic M. Concise review: mesenchymal stem cell treatment of the complications of diabetes mellitus. Stem Cells. 2011; 29:5–10. 10.1002/stem.556 21280154 PMC3059410

[11] Yan J, Tie G, Xu TY, Cecchini K, Messina LM. Mesenchymal stem cells as a treatment for peripheral arterial disease: current status and potential impact of type II diabetes on their therapeutic efficacy. Stem Cell Rev. 2013; 9:360–72. 10.1007/s12015-013-9433-8 23475434 PMC3683101

[12] Hanahan D, Coussens LM. Accessories to the crime: functions of cells recruited to the tumor microenvironment. Cancer Cell. 2012; 21:309–22. 10.1016/j.ccr.2012.02.022 22439926

[13] Rodriguez R, Rubio R, Menendez P. Modeling sarcomagenesis using multipotent mesenchymal stem cells. Cell Res. 2012; 22:62–77. 10.1038/cr.2011.157 21931359 PMC3351912

[14] Kennedy SR, Loeb LA, Herr AJ. Somatic mutations in aging, cancer and neurodegeneration. Mech Ageing Dev. 2012; 133:118–26. 10.1016/j.mad.2011.10.009 22079405 PMC3325357

[15] Newman AB, Yanez D, Harris T, Duxbury A, Enright PL, Fried LP, and Cardiovascular Study Research Group. Weight change in old age and its association with mortality. J Am Geriatr Soc. 2001; 49:1309–18. 10.1046/j.1532-5415.2001.49258.x 11890489

[16] Losonczy KG, Harris TB, Cornoni-Huntley J, Simonsick EM, Wallace RB, Cook NR, Ostfeld AM, Blazer DG. Does weight loss from middle age to old age explain the inverse weight mortality relation in old age? Am J Epidemiol. 1995; 141:312–21. 10.1093/aje/141.4.312 7840109

[17] Alley DE, Metter EJ, Griswold ME, Harris TB, Simonsick EM, Longo DL, Ferrucci L. Changes in weight at the end of life: characterizing weight loss by time to death in a cohort study of older men. Am J Epidemiol. 2010; 172:558–65. 10.1093/aje/kwq168 20682520 PMC3025636

[18] Andres R, Muller DC, Sorkin JD. Long-term effects of change in body weight on all-cause mortality. A review. Ann Intern Med. 1993; 119:737–43. 10.7326/0003-4819-119-7_Part_2-199310011-00022 8363208

[19] Huang SC, Wu JF, Saovieng S, Chien WH, Hsu MF, Li XF, Lee SD, Huang CY, Huang CY, Kuo CH. Doxorubicin inhibits muscle inflammation after eccentric exercise. J Cachexia Sarcopenia Muscle. 2017; 8:277–84. 10.1002/jcsm.12148 27897404 PMC5377412

[20] Camus V, Lanic H, Kraut J, Modzelewski R, Clatot F, Picquenot JM, Contentin N, Lenain P, Groza L, Lemasle E, Fronville C, Cardinael N, Fontoura ML, et al. Prognostic impact of fat tissue loss and cachexia assessed by computed tomography scan in elderly patients with diffuse large B-cell lymphoma treated with immunochemotherapy. Eur J Haematol. 2014; 93:9–18. 10.1111/ejh.12285 24520908

[21] Szulc P, Munoz F, Marchand F, Chapurlat R, Delmas PD. Rapid loss of appendicular skeletal muscle mass is associated with higher all-cause mortality in older men: the prospective MINOS study. Am J Clin Nutr. 2010; 91:1227–36. 10.3945/ajcn.2009.28256 20237137

[22] Browner WS, Seeley DG, Vogt TM, Cummings SR, and Study of Osteoporotic Fractures Research Group. Non-trauma mortality in elderly women with low bone mineral density. Lancet. 1991; 338:355–58. 10.1016/0140-6736(91)90489-C 1677708

[23] Toss F, Wiklund P, Nordström P, Nordström A. Body composition and mortality risk in later life. Age Ageing. 2012; 41:677–81. 10.1093/ageing/afs087 22820447

[24] Tidball JG. Regulation of muscle growth and regeneration by the immune system. Nat Rev Immunol. 2017; 17:165–78. 10.1038/nri.2016.150 28163303 PMC5452982

[25] Spalding KL, Bhardwaj RD, Buchholz BA, Druid H, Frisén J. Retrospective birth dating of cells in humans. Cell. 2005; 122:133–43. 10.1016/j.cell.2005.04.028 16009139

[26] Lynch M. Evolution of the mutation rate. Trends Genet. 2010; 26:345–52. 10.1016/j.tig.2010.05.003 20594608 PMC2910838

[27] Dvorak HF. Tumors: wounds that do not heal. Similarities between tumor stroma generation and wound healing. N Engl J Med. 1986; 315:1650–59. 10.1056/NEJM198612253152606 3537791

[28] Witsch E, Sela M, Yarden Y. Roles for growth factors in cancer progression. Physiology (Bethesda). 2010; 25:85–101. 10.1152/physiol.00045.2009 20430953 PMC3062054

[29] Wolf D, Reinhard A, Seckinger A, Katus HA, Kuecherer H, Hansen A. Dose-dependent effects of intravenous allogeneic mesenchymal stem cells in the infarcted porcine heart. Stem Cells Dev. 2009; 18:321–29. 10.1089/scd.2008.0019 18435573

[30] Albersen M, Fandel TM, Lin G, Wang G, Banie L, Lin CS, Lue TF. Injections of adipose tissue-derived stem cells and stem cell lysate improve recovery of erectile function in a rat model of cavernous nerve injury. J Sex Med. 2010; 7:3331–40. 10.1111/j.1743-6109.2010.01875.x 20561166 PMC3885341

[31] Matthews DR, Hosker JP, Rudenski AS, Naylor BA, Treacher DF, Turner RC. Homeostasis model assessment: insulin resistance and β-cell function from fasting plasma glucose and insulin concentrations in man. Diabetologia. 1985; 28:412–19. 10.1007/BF00280883 3899825

