# Supplementary material for: Can mesenchymal stem cell lysate reverse aging?
Source: Aging (Albany NY). 2018 Oct 24;10(10):2900–10. doi: 10.18632/aging.101595 (PMC6224235; doi:10.18632/aging.101595)
Supplement: Supplementary Figures [file aging-10-101595-s001.pdf]

SUPPLEMENTARY FIGURES

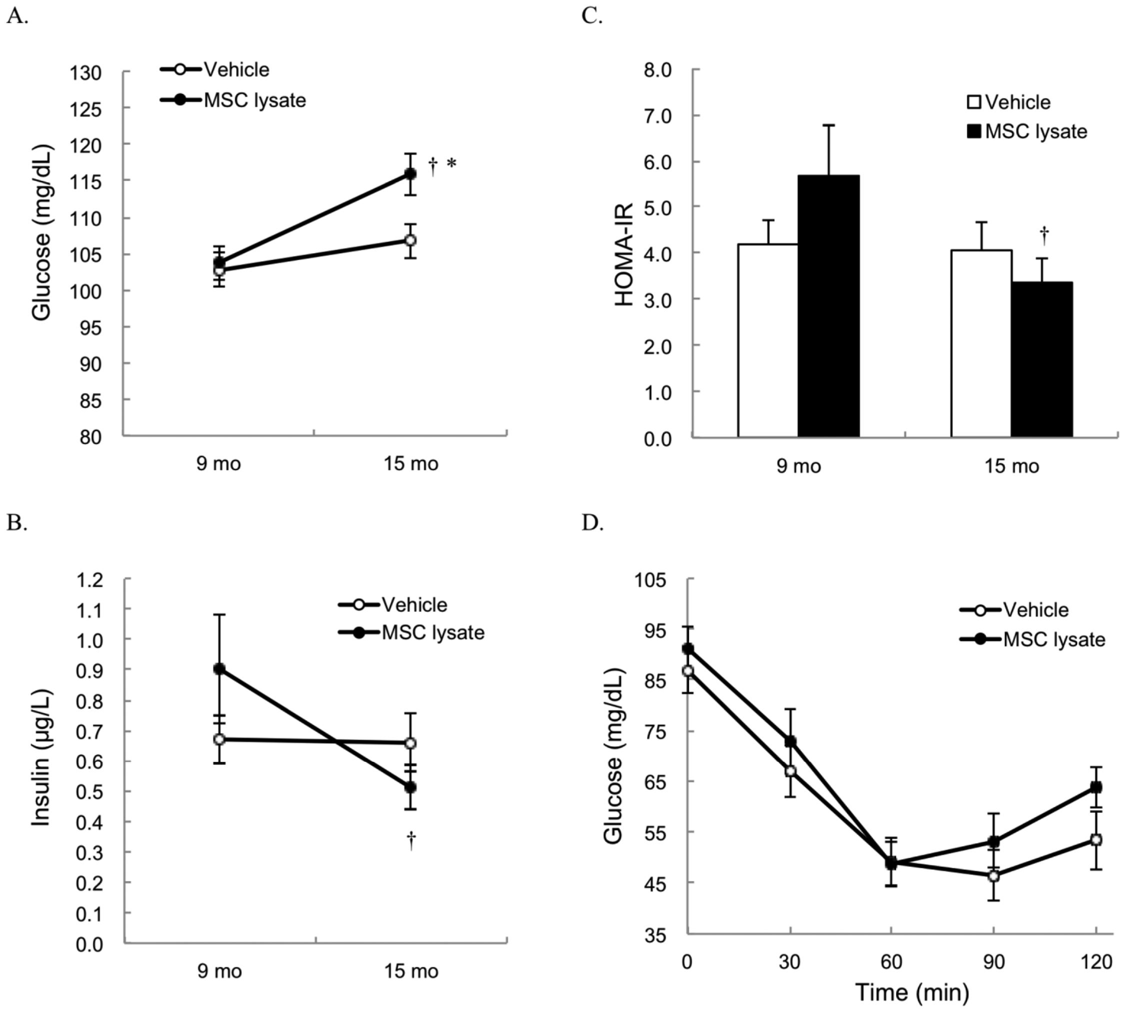

Figure S1_Male. Fasting glucose and insulin in blood, related to Figure 1. Male survivors until 15 months of age were included for the Pre-Post comparison (Vehicle: N = 22; MSC lysate: N = 19), measured at 9 months and 15 months of age. MSC lysate treatment increased fasting glucose (**A**) and decreased insulin (**B**), resulting in decreased HOMA-IR (**C**) after 2 sessions of MSC lysate treatment. No difference in blood glucose concentrations during insulin tolerance test (insulin injection at 0.3 U/kg body weight) were observed between the Vehicle and MSC lysate groups after 5 sessions of MSC lysate treatment (Vehicle: N = 13; MSC lysate: N = 9) (**D**). * Significant difference against the Vehicle group, $p \leq 0.05$. † Significant difference against pre-treatment value (9 months of age), $p \leq 0.05$. Abbreviation: MSC, adipose-derived mesenchymal stem cell.

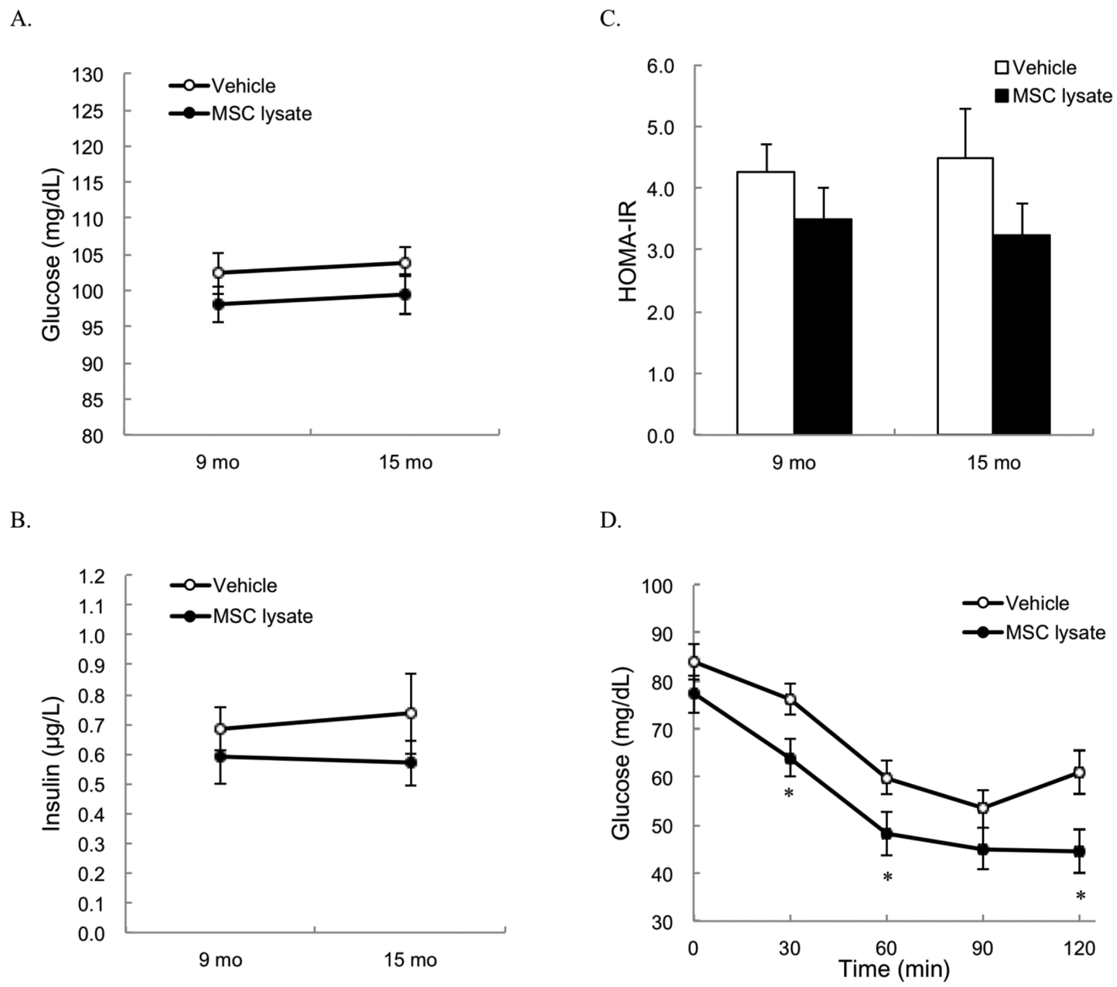

Figure S1_Female. **Fasting glucose and insulin in blood, related to Figure 1.** Female survivors until 15 months of age were included for the Pre-Post comparison (Vehicle: N = 21; MSC lysate: N = 20), measured at 9 months and 15 months of age. No significant differences in glucose (**A**), insulin (**B**), and HOMA-IR (**C**) were observed between the Vehicle and MSC lysate groups after 2 sessions of treatment. Blood glucose concentrations during insulin tolerance test (insulin injection at 0.3 U/kg body weight) of the MSC lysate group was significantly lower than those of the Vehicle group, after 5 sessions of MSC lysate treatment (Vehicle: N = 14; MSC lysate: N = 15) (**D**). * Significant difference against the Vehicle group, $p \leq 0.05$. Abbreviation: MSC, adipose-derived mesenchymal stem cell.

Log Rank, $p < 0.05$

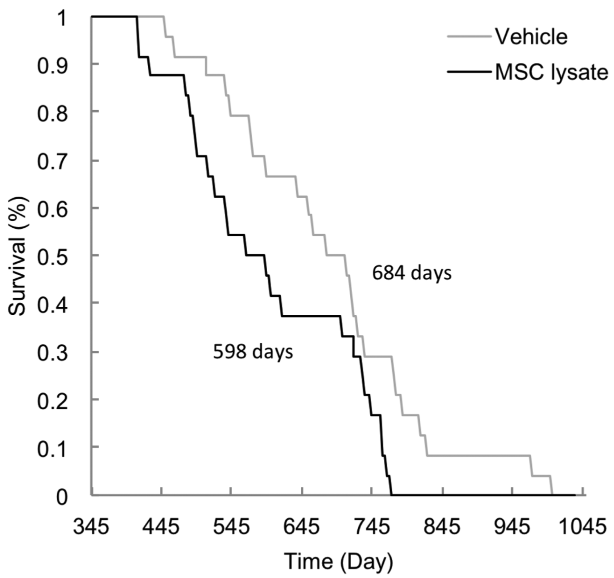

Figure S2-Male. **Survival curve of male rats, related to Figure 2.** MSC lysate treatment started from 12 months of age (Vehicle: N = 24; MSC lysate: N = 24). Mean lifespans of the vehicle and MSC lysate groups were 684 d and 598 d, respectively ($p \leq 0.05$). Abbreviation: MSC, adipose-derived mesenchymal stem cell.

Log Rank, $p = 0.32$

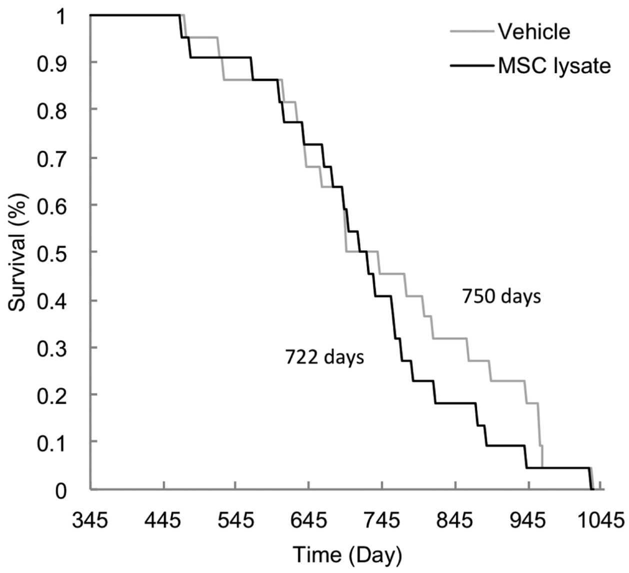

Figure S2_Female. **Survival curve of female rats, related to Figure 2.** MSC lysate treatment started from 12 months of age (Vehicle: N = 22; MSC lysate: N = 22). Mean lifespans of the vehicle and MSC lysate groups were 750 d and 722 d, respectively (*no significance*). Abbreviation: MSC, adipose-derived mesenchymal stem cell.

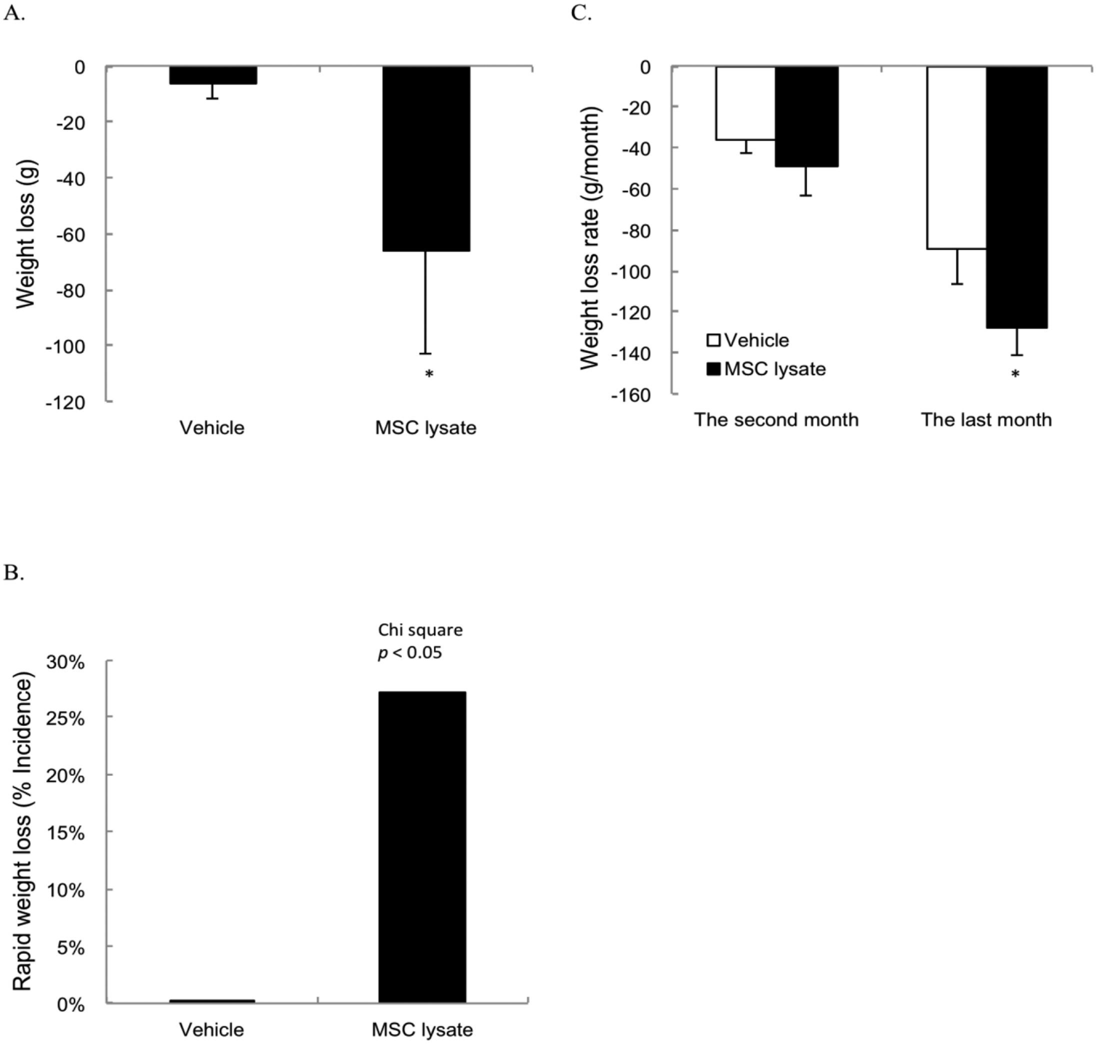

Figure S3_Male. Body weight changes, related to Figure 3. Male survivors until 15th months were included for the Pre-Post comparison (Vehicle: N = 22; MSC lysate: N = 19), measured at 9 months and 15 months of age. Significant weight loss was observed in the MSC lysate group following 2 sessions of intervention in the first 3 months ($p \leq 0.05$) (**A**). MSC lysate-treated rats showed greater incidence of rapid weight loss (more than 7% of peak body weight during their lifetime) than the Vehicle-treated rats (Chi-square, $p \leq 0.05$) (**B**). MSC lysate-treated rats had greater weight loss occurred 1 month before death compared with the Vehicle-treated rats (Vehicle: N = 24; MSC lysate: N = 24) (**C**). * Significant difference against the Vehicle group, $p \leq 0.05$. Abbreviation: MSC, adipose-derived mesenchymal stem cell.

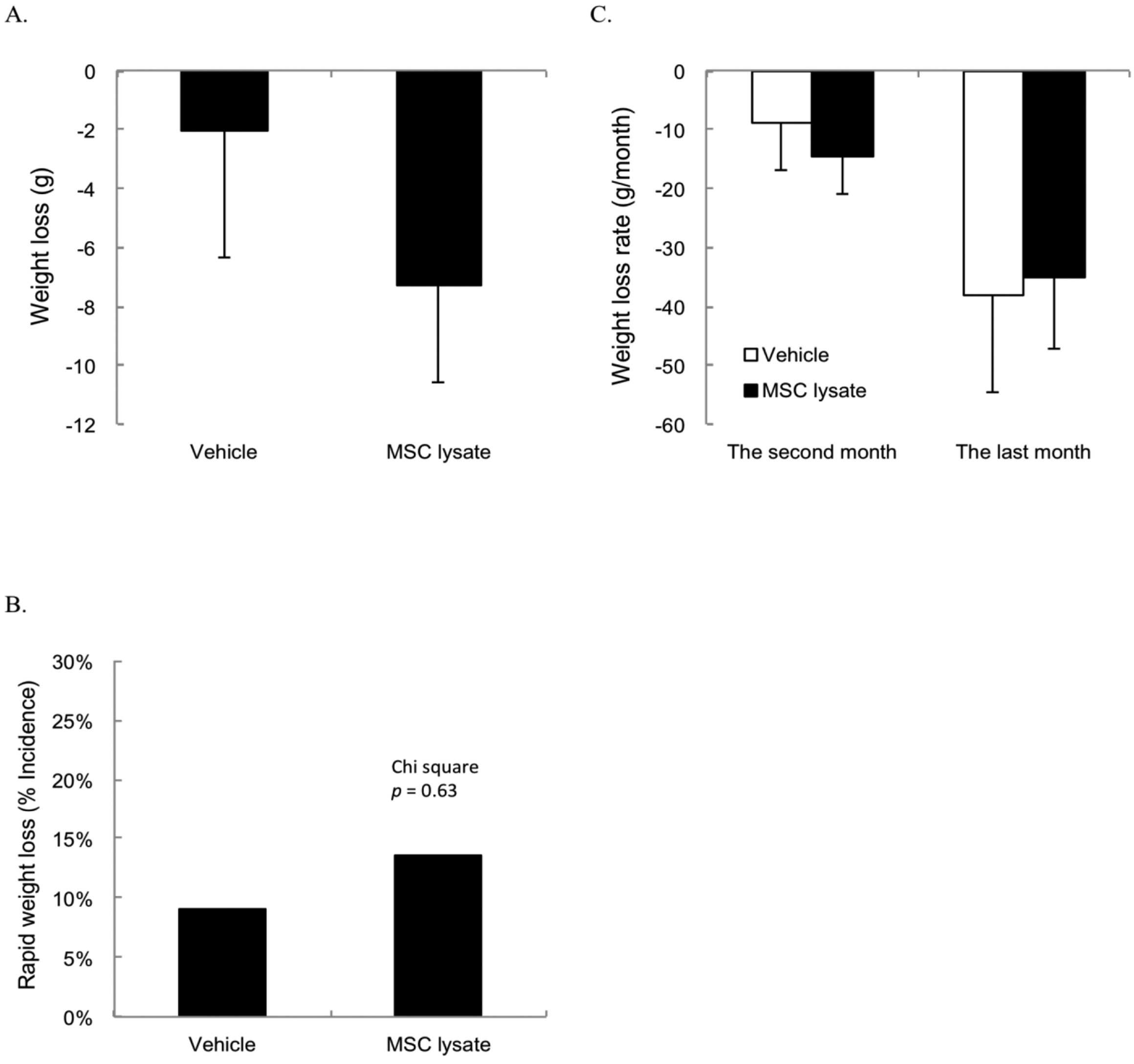

Figure S3_Female. Body weight changes, related to Figure 3. Female survivors until 15th months were included for the Pre-Post comparison (Vehicle: N = 21; MSC lysate: N = 20), measured at 9 months and 15 months of age. No significant difference in weight loss (**A**) was observed between the Vehicle and MSC lysate groups following 2 sessions of intervention. Similarly, no significant difference in the incidence of rapid weight loss (more than 7% of peak body weight during their lifetime) was observed between the Vehicle and MSC lysate groups after treatment (**B**). The magnitude of accelerated weight loss occurred 1 month before death was similar between both groups (Vehicle: N = 22; MSC lysate: N = 22) (**C**). Abbreviation: MSC, adipose-derived mesenchymal stem cell.
